# QuEST and High Performance Simulation of Quantum Computers

**DOI:** 10.1038/s41598-019-47174-9

**Published:** 2019-07-24

**Authors:** Tyson Jones, Anna Brown, Ian Bush, Simon C. Benjamin

**Affiliations:** 1 Department of Materials, University of Oxford, Parks Road, Oxford, OX1 3PH United Kingdom; 2 Oxford e-Research Centre, Department of Engineering Science, University of Oxford, Keble Road, Oxford, OX1 3PH United Kingdom

**Keywords:** Quantum simulation, Software

## Abstract

We introduce QuEST, the Quantum Exact Simulation Toolkit, and compare it to ProjectQ, qHipster and a recent distributed implementation of Quantum++. QuEST is the first open source, hybrid multithreaded and distributed, GPU accelerated simulator of universal quantum circuits. Embodied as a C library, it is designed so that a user’s code can be deployed seamlessly to any platform from a laptop to a supercomputer. QuEST is capable of simulating generic quantum circuits of general one and two-qubit gates and multi-qubit controlled gates, on pure and mixed states, represented as state-vectors and density matrices, and under the presence of decoherence. Using the ARCUS and ARCHER supercomputers, we benchmark QuEST’s simulation of random circuits of up to 38 qubits, distributed over up to 2048 compute nodes, each with up to 24 cores. We directly compare QuEST’s performance to ProjectQ’s on single machines, and discuss the differences in distribution strategies of QuEST, qHipster and Quantum++. QuEST shows excellent scaling, both strong and weak, on multicore and distributed architectures.

## Introduction

Quantum computation is a radical new paradigm of computing which takes advantage of the quantum phenomena seen at the microscopic physical scale. While significantly more challenging to engineer, quantum computers can run specialised algorithms which scale better than their classical counterparts; in some cases, exponentially faster^1^! A quantum computer operates upon a register of qubits, which are the quantum extension of classical bits. While a classical bit is confined to a definite value of either 0 or 1, a qubit can exist in a continuous complex space between these values. That is, describing the state of an *n*-bit classical register requires *n* bits, but describing the state of an *n*-qubit quantum register requires 2^*n*^ complex numbers. Consequently, simulating a quantum computer using a classical machine is believed to be exponentially costly with respect to the number of qubits.

Despite this, classical simulation of quantum computation is vital for the study of new algorithms and architectures. As experimental researchers move closer to realising quantum computers of sufficient complexity to be useful, their work must be guided by an understanding of what tasks we can hope to perform. This in turn means we must explore an algorithm’s scaling, its robustness versus errors and imperfections, and the relevance of limitations of the underlying hardware. Because of these requirements simulation tools are needed on many different classical architectures; while a workstation may be sufficient for the initial stages of examining an algorithm, further study of scaling and robustness may require more powerful computational resources. Flexible, multi-platform supporting simulators of quantum computers are therefore essential.

Further it is important these simulations are very efficient since they are often repeated many times, for example to study the influence of many parameters, or the behaviour of circuits under noise. But it is expensive to exactly simulate a quantum system using a classical system, since a high-dimensional complex vector must be maintained with high fidelity. Both the memory requirements, and the time required to simulate an elementary circuit operation, grow exponentially with the number of qubits. A quantum computer of only 50 qubits is too large to be comprehensively simulated by our best classical computers^2^, and has already been surpassed by Google’s 72 qubit Bristlecone QPU^3^. To simulate quantum computers even of the size already experimentally realised, it is necessary that a classical simulator take full advantage of the performance optimisations possible of high performance classical computing.

It is also equally important that the research community have access to an ecosystem of simulators. Verification of complex simulations is a non-trivial task, one that is much eased by having the facility to compare the results of simulations performed by multiple packages.

QuEST is an instance the class of so-called “direct evolution” simulators, whereby all information of the full quantum state is precisely maintained throughout the simulation. There are many other classes of specialised simulators, which for example use tensor network contractions^4^ and Monte Carlo sampling^2^, with their own memory, runtime and precision tradeoffs. These are suitable for different families of circuits and when different kinds of information about the final quantum state are sought. An excellent summary of these tradeoffs can be found in ref.^5^.

The number of single compute node generic^6–8^ and specialised^4,9–11^ simulators is rapidly growing. However despite many reported distributed simulators^12–19^ and proposals for GPU accelerated simulators^16,20–23^, QuEST is the first open source simulator available to offer *both* facilities, and the only simulator to offer support on all hardware platforms commonly used in the classical simulation of quantum computation.

## Background

### Target Platforms and Users

Simulations of quantum computation are performed on a wide variety of classical computational platforms, from standard laptops to the most powerful supercomputers in the world, and on standard CPUs or on accelerators such as GPUs. Which is most suitable for the simulation of a given circuit will depend upon the algorithm being studied and the size of the quantum computer being modelled. To date this has resulted in a number of simulators which typically target one, or a small number, of these architectures. While this leads to a very efficient exploitation of a given architecture, it does mean that should a research project need to move from one architecture to another, for instance due to the need to simulate more qubits, a different simulation tool is required. This may require a complete rewrite of the simulation code, which is time consuming and makes verification across platforms difficult. In this article we describe QuEST which runs efficiently on *all* architectures typically available to a researcher, thus facilitating the seamless deployment of the researcher’s code. This universal support also allows the researcher to easily compare the performance of the different architectures available to them, and so pick that most suitable for their needed simulations.

In the rest of this section we shall examine the nature of the architectures that are available, cover briefly how codes exploit them efficiently, and show how QuEST, the universal simulator, compares with the more platform specific implementations.

### Simulator Optimisations

Classical simulators of quantum computation can make good use of several performance optimisations.

For instance, the data parallel task of modifying the state vector under a quantum operation can be sped up with single-instruction-multiple-data (SIMD) execution. SIMD instructions, like Intel’s advanced vector extensions (AVX), operate on multiple operands held in vector registers to concurrently modify multiple array elements^24^, like state vector amplitudes.

Task parallelism can be achieved through multithreading, taking advantage of the multiple cores found in modern CPUs. Multiple CPUs can cooperate through a shared NUMA memory space, which simulators can interface with through OpenMP^25^.

Simulators can defer the expensive exchange of data in a CPU’s last level cache (LLC) with main memory through careful data access; a technique known as cache blocking^26^. Quantum computing simulators can cache block by combining sequential operations on adjacent qubits before applying them, a technique referred to as *gate fusion*^15,16^. For instance, gates represented as matrices can be fused by computing their tensor product.

Machines on a network can communicate and cooperate through message passing. Simulators can partition the state vector and operations upon it between distributed machines, for example through MPI, to achieve both parallelisation and greater aggregate memory. Such networks are readily scalable, and are necessary for simulating many qubit circuits^16^.

With the advent of general-purpose graphical processing units (GPGPUs), the thousands of linked cores of a GPU can work to parallelise scientific code. Simulators can make use of NVIDIA’s compute unified device architecture (CUDA) to achieve massive speedup on cheap, discrete hardware, when simulating circuits of a limited size^22^. We mention too a recent proposal to utilise multi-GPU nodes for highly parallel simulation of many qubit quantum circuits^21^.

#### Single node

ProjectQ is an open-source quantum computing framework featuring a compiler targeting quantum hardware and a C++ quantum computer simulator behind a Python interface^27^. In this text, we review the performance of its simulator, which supports AVX instructions, employs OpenMP and cache blocking for efficient parallelisation on single-node shared-memory systems, and emulation to take computational shortcuts^28^.

QuEST is a new open source simulator developed in ISO standard conformant C^29^, and released under the open source MIT license. Both OpenMP and MPI based parallelisation strategies are supported, and they may be used together in a so-called hybrid strategy. This provides seamless support for both single-node, shared-memory and distributed systems. QuEST also employs CUDA for GPU acceleration, and offers the same interface on single-node, distributed and GPU platforms. Though QuEST does not use cache blocking, AVX or emulation, we find QuEST performs equally or better than ProjectQ on multicore systems, and can use its additional message-passing facilities for faster and bigger simulations on distributed memory architectures.

ProjectQ offers a high-level Python interface, but can therefore be difficult to install and run on supercomputing architectures, though containerisation may make this process easier in future^30,31^. Conversely, Quest is light-weight, stand-alone, and tailored for high-performance resources - its low-level C interface can be compiled directly to a native executable and run on personal laptops and supercomputers.

**Figure 1:**
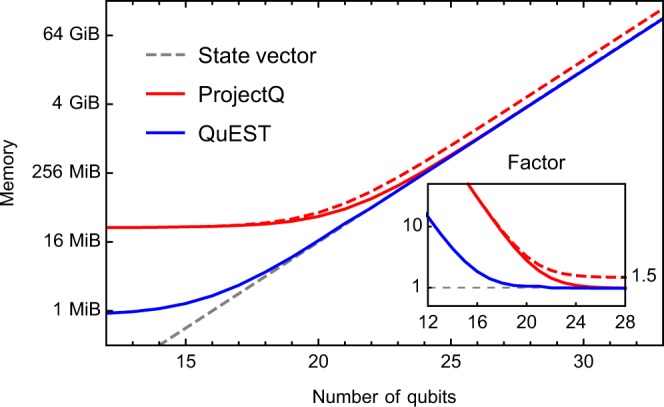
Memory consumption of QuEST’s C and ProjectQ’s Python processes, as reported by Linux’s/proc/self/status during random circuit simulation on a single 256 GiB ARCUS compute node. Full and dashed lines show the typical and maximum usage respectively, while the gray dashed line marks the memory required to store only the state-vector (in double precision). The subplot shows the ratio of total memory consumed to that by only the state-vector.

Both QuEST and ProjectQ maintain a pure state in 2^*n*^ complex floating point numbers for a system of *n* qubits, with (by default) double precision in each real and imaginary component; QuEST can otherwise be configured to use single or quad precision. Both simulators store the state in C/C++ primitives, and so (by default) consume 16 × 2^*n*^ B^32^ in the state vector alone. However ProjectQ incurs a × 1.5 memory overhead during state allocation, and QuEST clones the state vector in distributed applications. Typical memory costs of both simulators on a single thread are shown in Fig. 1, which vary insignificantly from their multithreaded costs. While QuEST allows direct read and write access to the state-vector, ProjectQ’s single amplitude fetching has a Python overhead, and writing is only supported in batch which is memory expensive due to Python objects consuming more memory than a comparable C primitive - as much as 3×^33,34^. Iterating the state-vector in ProjectQ is therefore either very slow, or comes with an appreciable memory cost, which may limit its usefulness in some simulation applications.

QuEST applies a general single-qubit gate (a 2 × 2 unitary matrix *G*) on qubit *q* of an *N*-qubit pure state-vector $|\psi \rangle =\sum _{n=0}^{{2}^{N}-1}\,{\alpha }_{n}|n\rangle$, represented as the complex vector $\overrightarrow{\alpha }$, by updating vector elements

1 $$(\begin{array}{l}{\alpha }_{{n}_{i}}\\ {\alpha }_{{n}_{i}+{2}^{q}}\end{array})\mapsto G(\begin{array}{l}{\alpha }_{{n}_{i}}\\ {\alpha }_{{n}_{i}+{2}^{q}}\end{array})$$

where ${n}_{i}=\lfloor i/{2}^{q}\rfloor {2}^{q+1}+(i\,{\rm{mod}}\,{2}^{q})$ for every integer $i\in [0,{2}^{N-1}-1]$. This applies *G* via 2^*N*^ computations of *ab* + *cd* for complex *a*, *b*, *c*, *d* and avoids having to compute and matrix-multiply a full 2^*N*^ × 2^*N*^ unitary on the state-vector. This lends itself to parallelisation and distribution, as described in the following section. In an effort to make efficient use of resources, QuEST features a number of bespoke functions to effect certain gates with fewer floating point operations than that involved in effecting general unitaries. For example, the Pauli-X operator $$(\begin{array}{ll}0 & 1\\ 1 & 0\end{array})$$ is implemented by swapping amplitudes without modifying them. Diagonal gates like the phase-shift, with matrix diag (1, exp(*iϕ*)), need only multiply half of the amplitudes with exp(*iϕ*) in an embarrassingly parallelisable manner. Multiple-controlled single-target unitaries with matrix representation diag (1, …, *G*) are effected by applying the 2 × 2 unitary matrix *G* to only a subset of the amplitudes otherwise modified when *G* is applied without any control qubits. Even multi-target unitaries of the form $\exp (i\varphi {\otimes }_{j}{Z}_{{q}_{j}})$ have an embarrassingly parallelisable implementation which multiplies each amplitude with exp(±*iϕ*) depending on the parity of the target qubits {*q*_*j*_}.

We leverage the same hardware-optimised code to enact gates on *N*-qubit density matrices, by storing them as 2*N*-qubit state-vectors,

2 $$\rho =\sum _{j=0}^{{2}^{N}-1}\,\sum _{k=0}^{{2}^{N}-1}\,{\alpha }_{j,k}|j\rangle \langle k|\to \rho ^{\prime} =\sum _{n=0}^{{2}^{2N}-1}\,{\alpha }_{n^{\prime} }|n\rangle .$$

Here the object *ρ*′ does not, in general, respect the constraint $\sum |{\alpha ^{\prime} }_{n}{|}^{2}=1$. An operation ${G}_{q}\rho {G}_{q}^{\dagger }$, that is a gate on qubit *q*, can then be effected on *ρ*′ as ${G}_{q+N}^{\ast }{G}_{q}\rho ^{\prime} ,$ by exploiting the Choi–Jamiolkowski isomorphism^35^. This holds also for multi-qubit gates. The distribution of the density matrix in this form lends itself well to the parallel simulation of dephasing, amplitude damping and depolarising noise channels.

#### Distributed

How simulators partition the state vector between processes and communicate over the network is key to their performance on distributed memory architectures. All simulators we have found so far employ a simple partitioning scheme; the memory to represent a state vector is split equally between all processes holding that vector. A common strategy to then evaluate a circuit is to pair nodes such that upon applying a single qubit gate, every process must send and receive the entirety of its portion of the state vector to its paired process^14,15,18^.

**Figure 2:**
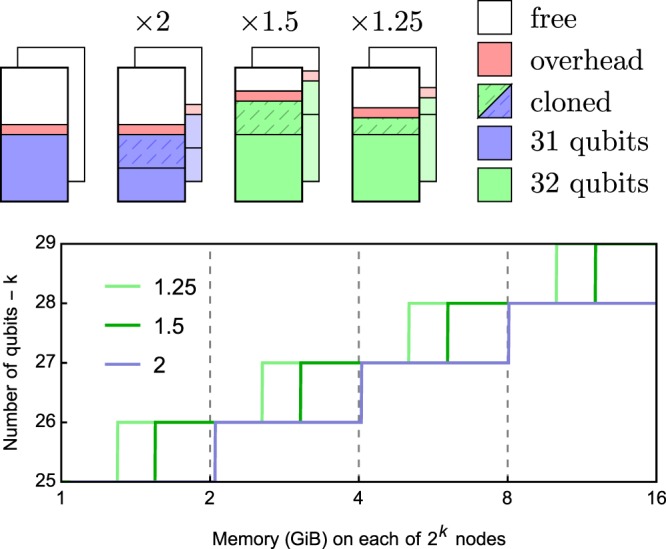
An illustration of strategies to distribute the state vector between two 64 GiB nodes. A complete cloning (×2 memory) of the partition on each node is wasteful. Half the partition can be cloned, at the cost of twice as many MPI messages, to fit another qubit into memory^14^. Further division requires more communication for less memory overhead^15^. The bottom plot shows the maximum number of qubits which can fit on 2^*k*^ nodes of varying memory, assuming a 50 MiB overhead per node.

The number of communications between paired processes, the amount of data sent in each and the additional memory incurred on the compute nodes form a tradeoff. A small number of long messages will ensure that the communications are bandwidth limited, which leads to best performance in the communications layer. However this results in a significant memory overhead, due to the process having to store buffers for both the data it is sending and receiving, and in an application area so memory hungry as quantum circuit simulation this may limit the size of circuit that can be studied. On the other hand many short messages will minimise the memory overhead as the message buffers are small, but will lead to message latency limited performance as the bandwidth of the network fabric will not be saturated. This in turn leads to poor parallel scaling, and hence again limits the size of the circuit under consideration, but now due to time limitations. Note that the memory overhead is at most a factor 2, which due to the exponential scaling of the memory requirements, means only 1 less qubit may be studied. Some communication strategies and their memory overheads and visualised in Fig. 2.

QuEST partitions the state vector equally between the processes within the job, and the message passing between the process pairs is so organised as to absolutely minimise the number of communications during the operation of a single gate. Thus parallel performance should be good, but there will be a significant memory overhead; in practice a factor of 2 as described above. For *n* qubits distributed over 2^*k*^ nodes, these communications occur when operating on qubits with index ≥*n* − *k*, indexing from 0.

An alternative strategy is to clone, send and receive only half of each node’s data in two exchanges^14^, incurring instead a 1.5× memory cost. This often leaves room to simulate an additional qubit, made clear in Fig. 2. This strategy can be recursed further to reduce the memory overhead even more, and negligible additional memory cost can be achieved by communicating every amplitude separately as in^18^, though this comes at a significant communication cost, since a message passing pattern is latency dominated and will exhibit poor scaling with process count. However an improvement made possible by having two exchanges is to overlap the communication of the first message with the computation on the second half of the state vector, an optimisation implemented in qHipster^15^. This depends on the network effectively supporting asynchronous communications.

We also mention recent strategies for further reducing network traffic by optimising the simulated circuit through gate fusion, state reordering^15,16^ and rescheduling operations^16^, though opportunities for such optimisations may be limited.

In terms of the functionality implemented in the simulation packages we note that while qHipster is limited to single and two-qubit controlled gates, QuEST additionally allows the distributed operation of any-qubit controlled gates.

#### GPU

Though MPI distribution can be used for scalable parallelisation, networks are expensive and are overkill for deep circuits of few qubits. Simulations limited to 29 qubits can fit into a 12 GB GPU which offers high parallelisation at low cost. In our testing, QuEST running a single Tesla K40m GPU (retailing currently for ~3.6 k USD) outperforms 8 distributed 12-core Xeon E5-2697 v2 series processors, currently retailing at ~21 k USD total, ignoring the cost of the network.

QuEST is the first available simulator of both state-vectors and density matrices which can run on a CUDA-enabled GPU, offering speedups of ~5× over already highly-parallelised 24-threaded single-node simulation. We mention some other single-node GPU-accelerated simulators under development such as QuantumSim; a CUDA-based simulator of density matrices^36^, Qrack; an OpenCL simulator of pure states^37^ and QCGPU^38^; a python wrapper of an OpenCL simulator though currently of limited functionality.

#### Multi-platform

QuEST is the only simulator which supports all of the above classical architectures. A simulation written in QuEST can be immediately deployed to all environments, from a laptop to a national-grade supercomputer, performing well at all simulation scales.

We list the facilities supported by other state-of-the-art simulators in Table 1.

**Table 1:** A comparison of the facilities offered by some publicly available, state-of-the-art simulators.

Simulator	multithreaded	distributed	GPU accelerated	stand-alone	density matrices
QuEST	✓	✓	✓	✓	✓
qHipster	✓	✓		✓	
QuantumSim			✓		✓
Qrack	✓		✓	✓	
ProjectQ	✓				

Here, density matrices refers to the ability to precisely represent mixed states. Note qHipster is no longer actively maintained, and has been renamed to Intel Quantum Simulator.

### Algorithm

**Figure 3:**
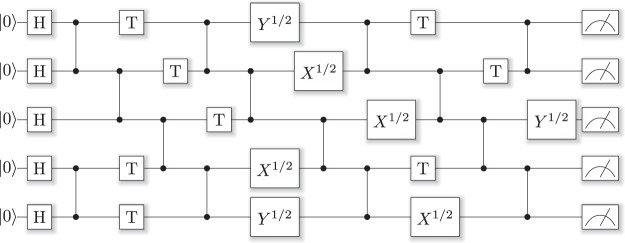
An example of a depth 10 random circuit on 5 qubits, of the linear topology described in^39^. This diagram was generated using ProjectQ’s circuit drawer.

We compare QuEST and ProjectQ performing simulations of universal psuedo-random quantum circuits of varying depth and number of qubits. A random circuit contains a random sequence of gates, in our case with gates from the universal set {*H*, *T*, *C*(*Z*), *X*^1/2^, *Y*^1/2^}. These are the Hadamard, *π*/8, controlled-phase and root Pauli X and Y gates. Being computationally hard to simulate, random circuits are a natural algorithm for benchmarking simulators^39^. We generate our random circuits by the algorithm in^39^, which fixes the topology for a given depth and number of qubits, though randomises the sequence of single qubit gates. An example is shown in Fig. 3. The total number of gates (single plus control) goes like ${\mathscr{O}}(nd)$ for an *n* qubit, depth *d* random circuit, and the ratio of single to control gates is mostly fixed at 1.2 ± 0.2, so we treat these gates as equal in our runtime averaging. For a sense of measurement, a depth 100 circuit of 30 qubits features 1020 single qubit gates and 967 controlled phase gates.

Though we here treat performance in simulating a random circuit as an indication of the general performance of the simulator, we acknowledge that specialised simulators may achieve better performance on particular classes of circuits. For example, ProjectQ can utilise topological optimisation or classical emulation to shortcut the operation of particular subcircuits, such as the quantum Fourier transform^28^.

We additionally study QuEST’s communication efficiency by measuring the time to perform single qubit rotations on distributed hardware.

## Setup

### Hardware

We evaluate the performance of QuEST and ProjectQ using Oxford’s computing facilities, specifically the ARCUS supercomputer, and the UK National Supercomputing facility ARCHER.

QuEST and ProjectQ are compared on single nodes with 1-16 threads, on ARCUS with nodes of 64, 128 and 256 GiB memory (simulating 1–31, 32 and 33 qubits respectively), each with two 8-core Intel Xeon E5-2640 V3 processors and a collective last level cache (LLC) size of 41 MB between two NUMA banks. We furthermore benchmark QuEST on ARCUS Tesla K40m GPU nodes, which with 12 GB global memory over 2880 CUDA cores, can simulate up to 29 qubit circuits.

QuEST and ProjectQ are also compared on ARCHER, a CRAY XC30 supercomputer. ARCHER contains both 64 and 128 GiB compute nodes, each with two 12-core Intel Xeon E5-2697 v2 series processors linked by two QuickPath Interconnects, and a collective LLC of 61 MB between two NUMA banks. Thus a single node is capable of simulating up to 32 qubits with 24 threads. We furthermore evaluate the scalability of QuEST when distributed over up to 2048 ARCHER compute nodes, linked by a Cray Aries interconnect, which supports an MPI latency of ~1.4 ± 0.1 *μ*s and a bisection bandwidth of 19 TB/s.

### Software

#### Installation

On ARCUS, we compile both single-node QuEST v0.10.0 and ProjectQ’s C++ backend with GCCZ5.3.0, which supports OpenMP 4.0^25^ for parallelisation among threads. For GPU use, QuEST is compiled with NVIDIA CUDA 8.0. ProjectQ v0.3.5 is run with Python 3.5.4, inside an Anaconda 4.3.8 environment.

On ARCHER, ProjectQ v0.3.6 is compiled with GCC 5.3.0, and run in Python 3.5.3 inside an Anaconda 4.0.6. QuEST is compiled with ICC 17.0.0 which supports OpenMP 4.5^25^, and is distributed with the MPICH3 implementation of the MPI 3.0 standard, optimised for the Aries interconnect.

#### Configuration

We attempt to optimise ProjectQ when simulating many qubits by enabling gate fusion only for multithreaded simulations^31^.
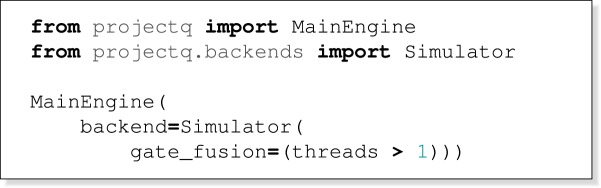


We found that ProjectQ’s multithreaded simulation of few qubit random circuits can be improved by disabling all compiler engines, to reduce futile time spent optimising the circuit in Python.
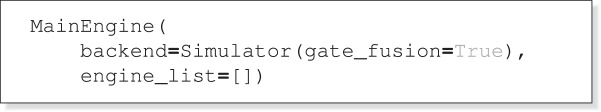


However, this disables ProjectQ’s ability to perform classical emulation and gate decomposition, and so is not explored in our benchmarking. We studied ProjectQ’s performance for different combinations of compiler engines, number of gates considered in local optimisation and having gate fusion enabled, and found the above configurations gave the best performance for random circuits on our tested hardware.

Our benchmarking measures the runtime of strictly the code responsible for simulating the sequence of gates, and excludes the time spent allocating the state vector, instantiating or freeing objects or other one-time overheads.

In ProjectQ, this looks like:
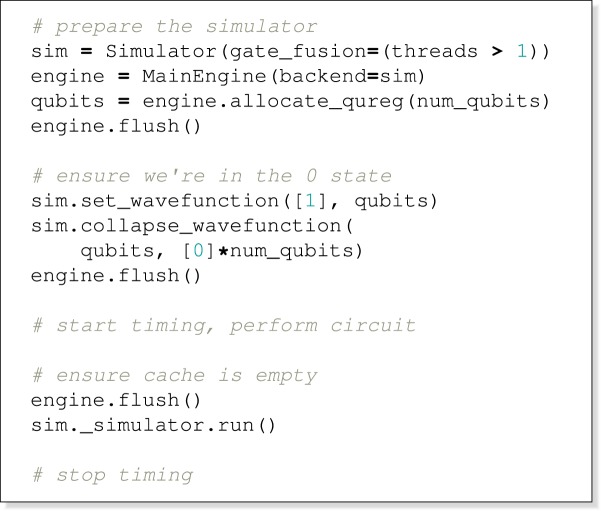


and in QuEST:
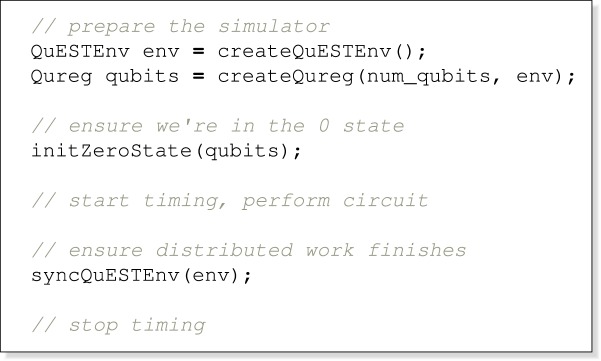


## Results

### Single node performance

**Figure 4:**
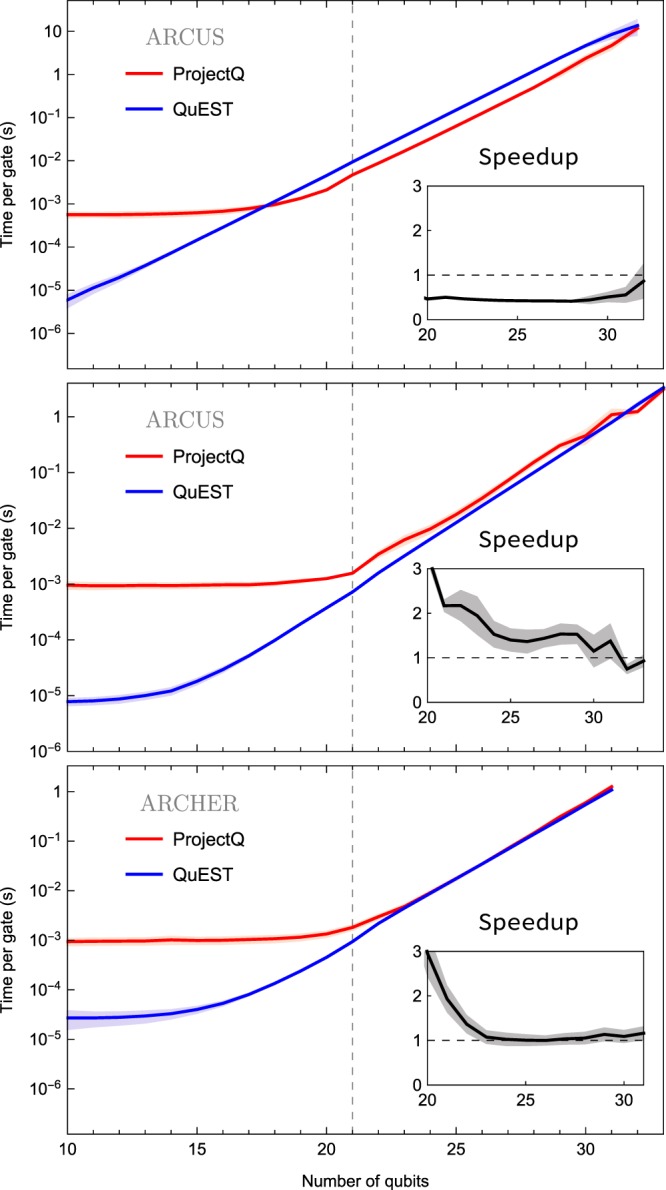
Comparison of QuEST and ProjectQ when simulating random circuits over 1, 16 (on ARCUS) and 24 (on ARCHER) threads (top to bottom). Coloured lines indicate the mean, with shaded regions indicating a standard deviation either side, over a total of ~77 k simulations of varying depth. Vertical dashed lines indicate the maximum number of qubits for which the entire state vector fits into the LLC. The speedup subgraphs show the ratio of ProjectQ to QuEST runtime.

The runtime performance of QuEST and ProjectQ, presented in Fig. 4, varies with the architecture on which they are run, and the system size they simulate. Anomalous slowdown of ProjectQ at 22 qubits may be explained by the LLC becoming full, due to its use of cache blocking through gate fusion^31^.

For fewer than ~22 qubits, ProjectQ’s Python overhead is several orders of magnitude slower than QuEST’s C overhead, independent of circuit depth. The Python overhead can be reduced by disabling some simulation facilities - see Section 3. For larger systems, the time spent in ProjectQ’s C backend operating on the state vector dominates total runtime, and the time per gate of both simulators grows exponentially with increasing number of qubits.

**Figure 5:**
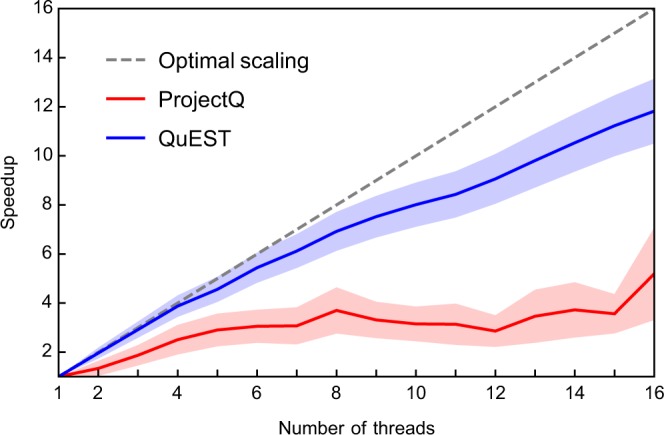
Single-node strong scaling achieved when parallelising (through OpenMP) 30 qubit random circuits across a varying number of threads on a 16-CPU ARCUS compute node. Solid lines and shaded regions indicate the mean and a standard deviation either side (respectively) of ~7 k simulations of circuit depths between 10 and 100.

On a single ARCUS-B thread, ProjectQ becomes twice as fast as QuEST, attributable to its sophisticated circuit evaluation. However, these optimisations appear to scale poorly; QuEST outperforms ProjectQ on 16 threads on ARCUS-B, and on ARCHER both simulation packages are equally fast on 24 threads. This is made explicit in the strong scaling over threads shown in Fig. 5, which reveals ProjectQ’s scaling is not monotonic. Performance suffers with the introduction of more than 8 threads, though is restored at 16.

**Figure 6:**
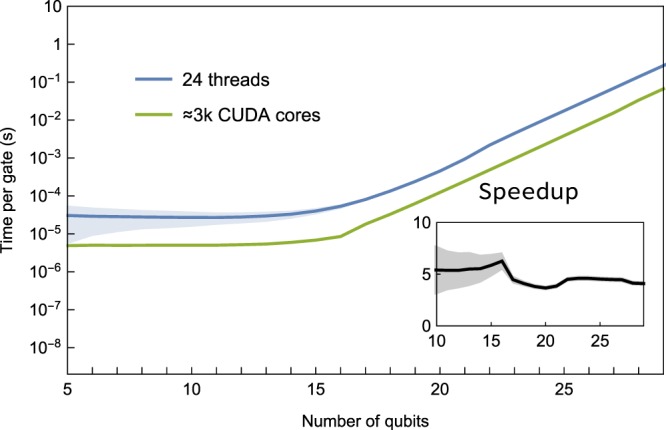
QuEST’s single-node performance using multithreading and GPU acceleration to parallelise random circuit simulations. The subplot shows the speedup (ratio of runtimes) that a GPU of 2880 CUDA cores on ARCUS achieves against 24 threads on ARCHER.

We demonstrate QuEST’s utilisation of a GPU for highly parallelised simulation in Fig. 6, achieving a speedup of ~5× from QuEST and ProjectQ on 24 threads.

### 0.1 Distributed Performance

**Figure 7:**
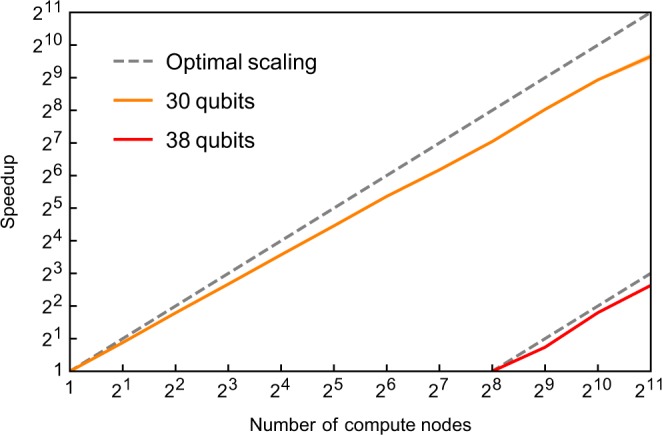
QuEST multinode strong scaling when distributing (through MPI) a depth 100 (depth 10) random circuit simulation of 30 qubits (38 qubits) across many 24-thread 64 GiB ARCHER nodes.

Strong scaling of QuEST simulating a 30 and 38 qubit random circuit, distributed over 1 to 2048 ARCHER nodes, is shown in Fig. 7. In all cases one MPI process per node was employed, each with 24 threads. Recall that QuEST’s communication strategy involves cloning the state vector partition stored on each node. The 30 qubit (38 qubit) simulations therefore demand 32 GiB (8 TiB) memory (excluding overhead), and require at least 1 node (256 nodes), whereas qHipster’s strategy would fit a 31 qubit (39 qubit) simulation on the same hardware^15^.

**Figure 8:**
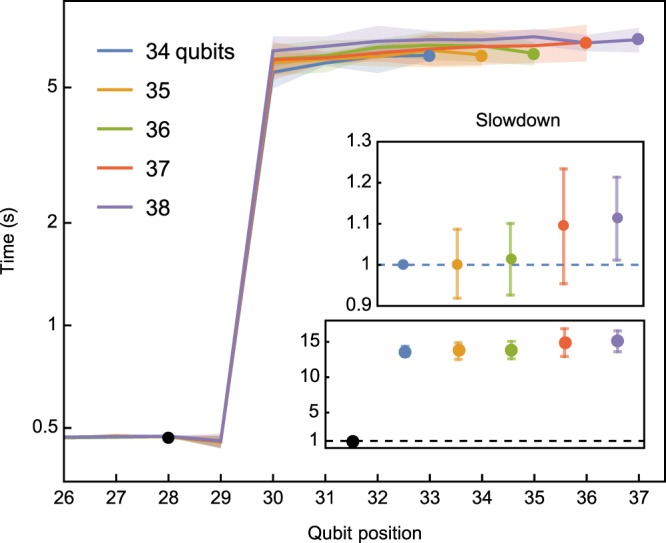
QuEST multinode weak scaling of a single qubit rotation, distributed on {16, 32, 64, 128, 256} ARCHER nodes respectively, each with 24 threads between two sockets and 64 GiB of memory. Communication occurs for qubits at positions ≥30, indexing from 0. Time to rotate qubits at positions 0–25 are similar to those of 26–29 and are omitted. The bottom subplot shows the slowdown caused by communication, while the top subplot shows the slowdown of rotating the final (communicated) qubit as the total number of qubits simulated increases from 34.

Communication cost is shown in Fig. 8 as the time to rotate a single qubit when using just enough nodes to store the state-vector; the size of the partition on each node is constant for increasing nodes and qubits. QuEST shows excellent weak scaling, and moving from 34 to 37 simulated qubits slows QuEST by a mere ≈9%. It is interesting to note that the equivalent results for qHipster show a slowdown of ≈148%^15^, but this is almost certainly a reflection of the different network used in generating those results, rather than in any inherent weakness in qHipster itself. QuEST and qHipster show comparable ~10^1^ slowdown when operating on qubits which require communication against operations on qubits which do not (shown in the bottom subplot of Fig. 8). Though such slowdown is also network dependent, it is significantly smaller than the ~10^6^ slowdown reported by the Quantum++ adaptation on smaller systems^18^, and reflects a more efficient communication strategy. We will discuss these network and other hardware dependencies further in future work, and also intend to examine qHipster on ARCHER so a true like with like comparison with QuEST can be made.

## Discussion

This paper introduced QuEST, a new high performance open source framework for simulating universal quantum computers. We demonstrated QuEST shows good strong scaling over OpenMP threads, competitive with a state of the art single-node simulator ProjectQ when performing multithreaded simulations of random circuits. We furthermore parallelised QuEST on a GPU for a 5× speedup over a 24 threaded simulation, and a 40× speedup over single threaded simulation. QuEST also supports distributed memory architectures via message passing with MPI, and we’ve shown QuEST to have excellent strong and weak scaling over multiple nodes. This behaviour has been demonstrated for up to 2048 nodes and has been used to simulate a 38 qubit random circuit. Despite its relative simplicity, we found QuEST’s communication strategy yields comparable performance to qHipster’s, and strongly outperforms the distributed adaptation of Quantum++. QuEST can be downloaded in ref.^40^.

## Data Availability

The code used to perform all benchmarking, and raw formats of the results themselves, are available in a public repository at https://github.com/QTechTheory/QuEST-ProjectQ-benchmarking. We note too that the two frameworks numerically tested in this work are open source and available at https://github.com/ProjectQ-Framework/ProjectQ and https://github.com/QuEST-Kit/QuEST.

## References

[1] Shor, P. W. Algorithms for quantum computation: discrete logarithms and factoring. In *Proceedings 35th Annual Symposium on Foundations of Computer Science*, 124–134, 10.1109/SFCS.1994.365700 (1994).

[2] Pednault, E. *et al*. Breaking the 49-qubit barrier in the simulation of quantum circuits arXiv:1710.05867 (2017).

[3] Villalonga, B. *et al*. A flexible high-performance simulator for the verification and benchmarking of quantum circuits implemented on real hardware arXiv:1811.09599 (2018).

[4] Fried, E. S. *et al*. qTorch: The quantum tensor contraction handler arXiv:1709.03636 (2017). 10.1371/journal.pone.0208510 PMC6287880 30532242

[5] Villalonga, B. *et al*. Establishing the quantum supremacy frontier with a 281 pflop/s simulation arXiv:1905.00444 (2019).

[6] Wecker, D. & Svore, K. M. LIQUij|>: a software design architecture and domain-specific language for quantum computing (2014).

[7] Smith, R. S., Curtis, M. J. & Zeng, W. J. A practical quantum instruction set architecture arXiv:1608.03355 (2016).

[8] Heston, K., Delimarsky, D., Geller, A. & Wecker, D. The Q# programming language (2017).

[9] Zulehner, A. & Wille, R. Advanced simulation of quantum computations arXiv:1707.00865 (2017).

[10] Bravyi S, Gosset D (2016). Improved classical simulation of quantum circuits dominated by clifford gates. Phys. Rev. Lett..

[11] Dahlberg A, Wehner S (2019). SimulaQron - a simulator for developing quantum internet software. Quantum Sci. Technol..

[12] De Raedt K (2007). Massively parallel quantum computer simulator. Comput. Phys. Commun..

[13] Niwa J, Matsumoto K, Imai H (2002). General-purpose parallel simulator for quantum computing. Phys. Rev. A.

[14] Trieu, D. B. Large-scale simulations of error-prone quantum computation devices. Dr. (univ.), Univ. Diss. Wuppertal, Jülich (2009). Record converted from VDB: 12.11.2012; Wuppertal, Univ. Diss. (2009).

[15] Smelyanskiy, M., Sawaya, N. P. D. & Aspuru-Guzik, A. qHiPSTER: The quantum high performance software testing environment arXiv:1601.07195 (2016).

[16] Häner, T. & Steiger, D. S. 0.5 petabyte simulation of a 45-qubit quantum circuit. In *Proceedings of the International Conference for High Performance Computing, Networking, Storage and Analysis*, SC ’17, 33:1–33:10, 10.1145/3126908.3126947 (ACM, New York, NY, USA, 2017).

[17] Khammassi, N., Ashraf, I., Fu, X., Almudever, C. G. & Bertels, K. QX: A high-performance quantum computer simulation platform. In *Design, Automation Test in Europe Conference Exhibition (DATE)*, **2017**, 464–469, 10.23919/DATE.2017.7927034 (2017).

[18] LaRose, R. Distributed memory techniques for classical simulation of quantum circuits arXiv:1801.01037 (2018).

[19] Chen Zhao-Yun, Zhou Qi, Xue Cheng, Yang Xia, Guo Guang-Can, Guo Guo-Ping (2018). 64-qubit quantum circuit simulation. Science Bulletin.

[20] Amariutei, A. & Caraiman, S. Parallel quantum computer simulation on the GPU. In *15th International Conference on System Theory, Control and Computing*, 1–6 (2011).

[21] Zhang, P., Yuan, J. & Lu, X. Quantum computer simulation on multi-GPU incorporating data locality. In Wang, G., Zomaya, A., Martinez, G. & Li, K. (eds) *Algorithms and Architectures for Parallel Processing*, 241–256 (Springer International Publishing, Cham, 2015).

[22] Gutiérrez E, Romero S, Trenas MA, Zapata EL (2010). Quantum computer simulation using the CUDA programming model. Comput. Phys. Commun..

[23] Savran, I., Demirci, M. & Yilmaz, A. H. Accelerating shor’s factorization algorithm on GPUs arXiv:1801.01434 (2018).

[24] Lomont, C. Introduction to Intel advanced vector extensions. *Intel white paper* (2011).

[25] OpenMP compilers & tools. http://www.openmp.org/resources/openmp-compilers/ Accessed: 2018-02-14 (2016).

[26] Lam MD, Rothberg EE, Wolf ME (1991). The cache performance and optimizations of blocked algorithms. SIGARCH Comput. Archit. News.

[27] Steiger DS, Häner T, Troyer M (2018). ProjectQ: an open source software framework for quantum computing. Quantum.

[28] Häner, T., Steiger, D. S., Svore, K. & Troyer, M. A software methodology for compiling quantum programs. *Quantum Sci. Technol*. (2018).

[29] International standard - programming languages - C ISO/IEC 9899:1999. http://www.open-std.org/jtc1/sc22/wg14/www/standards (1999).

[30] Jones, T. Installing ProjectQ on supercomputers. https://qtechtheory.org/resources/installing_projectq_on_supercomputers Accessed 30-5-2018 (2018).

[31] Häner, T. private communication (2017).

[32] Fundamental types, C++ language reference, microsoft developer network. https://msdn.microsoft.com/en-us/library/cc953fe1.aspx. Accessed: 2018-2-05.

[33] Foundation, P. S. *Data model, the Python language reference*. https://docs.python.org/3/reference/datamodel.html Accessed 20-5-2018 (2018).

[34] d’Informatique des Systémes Adaptatifs, L. Python memory management. http://deeplearning.net/software/theano/tutorial/python-memory-management.html Accessed 20-5-2018 (2017).

[35] Choi M-D (1975). Completely positive linear maps on complex matrices. Linear algebra its applications.

[36] Tarasinski, B., Ostroukh, V. & O’Brien, T. QuantumSim. https://github.com/quantumsim/quantumsim (2013).

[37] Strano, D. & Bollay, B. Qrack. https://github.com/vm6502q/qrack (2017).

[38] Kelly, A. Simulating quantum computers using OpenCL arXiv:1805.00988 (2018).

[39] Boixo, S. *et al*. Characterizing quantum supremacy in near-term devices arXiv:1608.00263 (2016).

[40] QuEST: The quantum exact simulation toolkit. https://quest.qtechtheory.org (2018).

